# Thermoring Basis for the Heat Inactivation in TRPV1

**DOI:** 10.21203/rs.3.rs-3280283/v1

**Published:** 2023-08-25

**Authors:** Guangyu Wang

**Affiliations:** University of California Davis

**Keywords:** cyclization against decyclization, desensitization, grid thermodynamics, lipid, non-covalent interaction, noxious heat damage, systemic thermal instability, thermorings, thermosensitivity, threshold

## Abstract

Transient receptor potential vanilloid-1 (TRPV1) in mammals exhibits the temperature-dependent inactivation in response to repeated or constant heat stimuli. However, the underlying structural factors or motifs are unresolved. In this computational study, the graph theory-based grid thermodynamic model was employed to reveal how the temperature-dependent non-covalent interactions as identified in the 3D structures of TRPV1 could develop a systemic fluidic grid-like mesh network with topological grids constrained as the thermo-rings to govern the heat inactivation from open and pre-open closed states in different temperature ranges. The results showed that the heat-evoked melting of three biggest grids in different gating states was responsible for the TRPV1 activity starting at 43°C and peaking at 56°C and ending at 61°C. While the second biggest grid controlled a reversible inactivation from the open state between56°C and 61°C, a smaller grid governed another irreversible inactivation from the pre-open closed state from 43°C to 61°C. Thus, two distinct inactivation pathways of TRPV1 may be involved in a protective mechanism in mammals against noxious heat.

## 1. Introduction

Transient receptor potential (TRP) vanilloid-1 (TRPV1) in mammals is characterized by the intriguing temperature-dependent inactivation in response to a heat stimulus. Following the activation at a start temperature threshold 43°C, its activity peaks at 56°C and then declines until 61°C [1–3]. Meanwhile, this thermosensitive channel has the overall use-dependent desensitization between 43°C and 61°C [1, 3, 4], which is coupled to the heat activation and impedes activation by capsaicin [3]. In contrast, TRPV3 displays the use-dependent sensitization upon repeated heat stimuli [5–11]. After an exchange of the C-terminus between rat TRPV1 (rTRPV1) and mouse TRPV3 (mTRPV3) was reported to eliminate the use-dependent heat desensitization found in rTRPV1 [12], the thermosensitive dynamic interaction between the N- and C-termini (segments 1–433 and 688–839, respectively) of mouse TRPV1 (mTRPV1) rather than platypus (*Orni-thorhynchus anatinus*) TRPV1 (pTRPV1) has been indicated to trigger a conformational rearrangement in the outer pore causing desensitization [4]. Furthermore, such desensitization is necessary to prevent cell swelling/death of TRPV1-expressing cells and thus to lower the risk of scald injury in mammals [4]. Until now, although the temperature-dependent 3D structures of capsaicin-bound rTRPV1 at low and high temperatures have been available [13], much less is known about the structural factors or motifs for the temperature-dependent inactivation.

Recently, following the report that thermal unfolding of a DNA hairpin is controlled by not only the strength of the H-bond in the stem but also the loop length [14], a graph theory-based grid thermodynamic model has been developed to reveal the thermoring basis for the thermal activation and inactivation of biomacromolecules such as class I and II fructose aldolases [15–17]. Briefly speaking, once noncovalent interactions in a single polypeptide chain form a systematic fluid grid-like mesh network, topological grids can be constrained as the thermorings from the biggest grid to the smallest one. If the calculated melting temperature threshold $\left(T_{m}\right)$ of a thermoring matches the temperature threshold $\left(T_{th}\right)$ for activation or inactivation of protein, that thermoring can be defined as the underlying structural factor or motif. In this computational study, the same strategy was used to define the structural motif for the inactivation of TRPV1 from the open state in a temperature range from 56°C to 61°C.

On the other hand, the melting of the biggest grid inevitably causes a change in the whole systematic fluidic noncovalent interaction mesh network including the smaller grids for the final heat efficacy. Thus, thermal unfolding of the biggest grid of TRPV1 is also required to release the inhibitive smaller grid in a closed state at a higher calculated $T_{m}$. In this regard, the inactivating thermoring below the $T_{m}$ of the biggest grid can be identified in TRPV1. Further, once the structural thermo-sensitivity $\left(\Omega_{10}\right)$ was defined as the change in the chemical potential of the total grids upon the change in the total enthalpy included in the noncovalent interactions as identified in the high-resolution 3D structures of an ion channel, it has been shown as comparable to the functional thermo-sensitivity $\left(Q_{10}\right)$ (the ratio of rates or open probabilities $\left(P_{o}\right)$ of an ion channel measured 10°C apart) of rat or human TRPV1 (rTRPV1 or hTRPV1, respectively) [18]. Therefore, with the open state being a control, the $\Omega_{10}$ value, together with the grid-based systematic thermal instability $\left(T_{i}\right)$ could be used in this investigation to identify different inactivation pathways in TRPV1 [15–18].

## 2. Methods

### 2.1 Data mining resources

The systemic fluidic grid-like non-covalently interacting mesh networks of rTRPV1 in different gating states were based on the cryo-electronic microscopy (cryo-EM) structural data of closed rTRPV1 in MSP2N2 with PI-bound at 4°C (PDB ID, 7LP9, modelresolution=2.63Å) and 48°C (PDB ID, 7LPC, modelresolution=3.07Å), capsaicin (Cab)-bound at 4°C (PDB ID, 7LPA, modelresolution=3.37Å), 25°C (PDB ID, 7LPB, modelresolution=3.54Å), and 48°C for 10 s (EMD ID, 23477, modelresolution=3.70Å) and 30 s (EMD 23478; PDB ID, 7LPD, modelresolution=3.55Å) [13]. Meanwhile, open rTRPV1 with Cap-bound at 48°C (PDB ID, 7LPE, modelresolution=3.72Å) and the the pre-open state of rTRPV1 with resiniferatoxin (RTX) bound at 25°C (PDBID, 7RQX, modelresolution=3.73Å were used as important controls [13, 19].

### 2.2 The definition of the necessary gating pathway

The phosphatidylinositol (PI)-dependent gating pathway from D388 in the pre-S1 domain to K710 in the TRP domain has been successfully defined to identify the thermorings for the matched temperature thresholds and sensitivity and systematic thermal instability $\left(T_{i}\right)$ of rTRPV1 [18]. Therefore, this PI-dependent gating pathway was also used in this study to identify the thermorings for the heat inactivation from both open and pre-open closed states unless the search for the biggest grid beyond this PI-dependent gating pathway was necessary.

### 2.3 Standards to filter non-covalent interactions

UCSF Chimera as well as the same strict and consistent standard definition as described and examined previously were employed as a filter to scan the stereo- or regio-selective inter-domain diagonal and intra-domain lateral non-covalent interactions along the PI-dependent gating pathway of rTRPV1 (Tables S1–5) [15–18]. They included salt-bridges, lone pair/CH/cation-π interactions and H-bonds between amino acid side chains. Of special note, momentary fluctuation-induced perturbations in the noncovalent interactions during protein dynamics were not considered.

### 2.4 Preparation of topological grid maps by using graph theory

**Figure 1:**
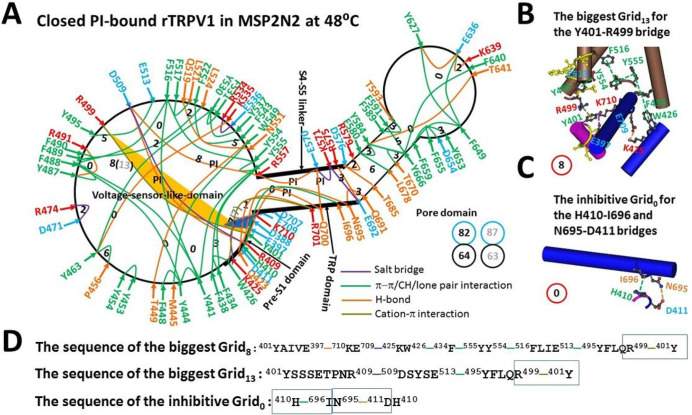
The grid-like non-covalently interacting mesh network along the PI-dependent gating pathway of PI-bound rTRPV1 in the closed state at 48 °C. **A,** The topological grids in the systemic fluidic grid-like mesh network. The cryo-EM structure of one subunit in closed rTRPV1 with PI bound at 48 °C (PDB ID, 7LPC) was used for the model. The pore domain, the S4-S5 linker, the TRP domain, the VSLD and the pre-S1 domain are indicated in black. Salt bridges, p interactions, and H-bonds between pairing amino acid side chains along the PI-dependent gating pathway from D388 to K710 are marked in purple, green, and orange, respectively. The grid sizes required to control the relevant non-covalent interactions were calculated with graph theory and labeled in black. The Y401-R499 bridge in the biggest Grid_13_ was highlighted in yellow. The total grid sizes and grid size-controlled non-covalent interactions along the PI-dependent gating pathway are shown in the cyan and black circles, respectively. When the inhibitive E397-K710 H-bond, which was highlighted in blue, was removed in the native lipid system, the putative grid sizes are labeled in grey. **B,** The structure of the biggest Grid_8_ with an 8-atom size for the Y401-R499 cation-p interaction near PI. **C,** The structure of the inhibitive Grid_0_ with a 0-residue size in the TRP/pre-S1 interface to control the H410-I696 and D411-N695 bridges near PI. **D,** The sequences of the biggest Grid_8_, Grid_13_ and Grid_0_ with 8-, 13- and 0-residue sizes to control the relevant bridges in the blue rectangles, respectively.

Once the non-covalent interactions were filtered, all the grids as defined previously were geometrically mapped along the PI-dependent gating pathway in the different gating states and at distinct temperatures [15–18]. The grid size (S) was constrained by the Floyd-Warshall algorithm as the minimal number of the total side chains of residues in protein or atoms in the bound lipid that did not involve any non-covalent interaction in a grid [20]. As a consequence, the shortest direct or reverse path between two ends of a noncovalent interaction was available. For example, in the grid-like biochemical reaction mesh network of Fig. 1A, a direct path length from Y627 and E636 was zero because there was an H-bond between them. However, there was another shortest reverse path from E636 to F649 and back to Y627 via two π interactions. Because nothing but these three residues was involved in the non-covalent interactions, the grid size was zero. Once each non-covalent interaction was tracked with a grid size, the unshared sizes were then marked in black. Taken as a whole, a grid with an x-residue or atom size was denoted as ${Grid}_{x}$, and the total non-covalent interactions and grid sizes along the PI-dependent gating pathway of one subunit were calculated and shown in black and cyan circles beside the mesh network map, respectively, in favor of the calculation of the systematic thermal instability $\left(T_{i}\right)$ and the structural thermosensitivity $\left(\Omega_{10}\right)$.

### 2.5 Equations

Following the report that thermal unfolding of the DNA hairpin was controlled by the G-C base pairs or the H-bonds in the stem and the loop length [14], the same equation as described and examined previously was used to calculate the melting temperature threshold $\left(T_{m}\right)$ for thermal unfolding of the given grid [15–18]:

(1) $${\text{T}}_{\text{m}}\left({}^{{}^{\circ}}\text{C}\right)=34\;+\;(\text{n}\;-\;2)\;\times \;10\;+\;\left(20\;-\;{\text{S}}_{\max}\right)\;\times \;2$$

where, n is the total number of simple H-bonds energetically equivalent to the non-covalent interactions controlled by the given grid, and $S_{max}$ is the size of the given grid. Accordingly, a decrease in the grid size or an increase in equivalent H-bonds will raise the grid’s heat capacity.

Similarly, the same equation as described and examined previously was employed to define and to calculate the systematic thermal instability $\left(T_{i}\right)$ along the PI-dependent gating pathway [15–18]:

(2) $$T_{i}=S/N$$

where, S and N are the total grid sizes and the total non-covalent interactions along the PI-dependent gating pathway of one subunit in a given gating state. On the ground of this definition, the lower $T_{i}$ means the less conformational entropy in the system.

For enthalpy-driven TRPV1 opening from the pre-open closed state within a temperature range ΔT as a result of the broken biggest grid, if the chemical potential of a grid is theoretically defined as the maximal potential for equivalent residues in the grid to form the tightest β-hairpin with the smallest loop via non-covalent interactions [21], the grid-based structural thermo-sensitivity $\left(\Omega_{\Delta T}\right)$ of a single ion channel can be defined and calculated using the following equations:

(3) $$\Omega_{\Delta T}={\left[\left(S_{c}-S_{o}\right)E/2\right]}^{\left(Hc/Ho\right)}={\left[\left(S_{c}-S_{o}\right)E/2\right]}^{\left[(ENc)/(ENo)\right]}={\left[\left(S_{c}-S_{o}\right)E/2\right]}^{\left(Nc/No\right)}$$

where, along the same PI-dependent gating pathway of one subunit, $N_{c}$ and $N_{o}$ are the total non-covalent interactions, $H_{c}$ and $H_{o}$ are the total enthalpy included in them, and $S_{c}$ and $S_{o}$ are the total grid sizes in the closed and open states, respectively. E is the energy intensity of a non-covalent interaction in a range of 0.5–3 kJ/mol. Usually, E is 1 kJ/mol [22]. Thus, $\Omega_{\Delta T}$ factually reflects a thermo-evoked change in the total chemical potential of grids upon a thermo-evoked change in the total enthalpy included in the non-covalent interactions from a closed state to an open state along the same PI-dependent gating pathway of one subunit. For the enthalpy-driven TRPV1 inactivation from the pre-open state within a temperature range ΔT as a result of the broken biggest grid, the same equation was used to calculate the apparent $\Omega_{\Delta T}$ value between open and inactivated states after the closed state was replaced with the inactivated state.

When $\Delta T=10^{{}^{\circ}}C,\;\Omega_{10}$ could be comparable to the functional thermo-sensitivity $\left(Q_{10}\right)$ of a single ion channel. $Q_{10}$ during the thermal activation was calculated using the following equation:

(4) $$Q_{10}={\left(X_{2}/X_{1}\right)}^{10/(T2-T1)}$$

However, during the thermal inactivation, it was calculated using the following equation:

(5) $$Q_{10}=-{\left(X_{1}/X_{2}\right)}^{10/(T2-T1)}$$

where, $X_{1}$ and $X_{2}$ are open probability $\left(P_{0}\right)$ values or reaction rates obtained at temperatures T1 and T2 (measured in kelvin), respectively.

## 3. Results

### 3.1. Identification of an Inactivating Motif in Closed PI-bound rTRPV1 at 48°C

Recent study demonstrated that the presence of the E397-K710 and Y401-D707 H-bonds causes the PI pocket to be the biggest Grid_8_ when rTRPV1 is reconsituted in MSP2N2 [18]. In this case, the caculated $T_{m}$ of 56°C prevents the PI release from the active vanilloid site for channel opening even if the M1-D276 and N753-K838 segments have been melted at 48°C [13]. However, the recent high-resolution cryo-EM structure of hTRPV1 demonstrated that the equivalent Y402-D708 H-bond is broken when the hydroxyl group on Y402 is orientated away from the carboxyl group of D708 [23]. In that case, another biggest Grid_8_ was created to control the Y401-R499 π interaction in the pre-S1/VSLD interface. It had an 8-residue size via the shortest path from Y401 to E397, K710, E709, K425, W426, F434, Y555, Y554, F516, E513, Y495, R499 and back to Y401 (Fig. 1A, B & D). When 1.5 equivalent H-bonds sealed it, the calculated $T_{m}$ was 53°C (Table 1). Thus, even if the Y401-D707 H-bond were broken, rTRPV1 in MSP2N2 could not open at 48°C [13]. When the total noncovalent interactions and grid sizes were 64 and 82, respectively (Fig. 1A, Table S1), the calculated systematic thermal instability was about 1.28 (Table 1). When the same open state was used as a control [18], the calculated mean strucutral thermosensitivity $\left(\Omega_{10}\right)$ was about 12.7, which was still smaller than the experimental $Q_{10}$ of 21.9 for oocytes-expressing rTRPV1 (Table 1) [13].

Once the E397-K710 H-bond was broken in the native membrane, the same biggest Grid_13_ had a 13-residue size via the shortest path from Y401 to R409, D509, E513, Y495, R499 and back to Y401 to govern the same Y401-R499 π interaction (Fig. 1A & D). Thus, the predicted $T_{m}$ still matched the experimental $T_{\text{th}}$ of 43°C (Table 1) [1–2]. In that case, in direct line with the recent report [18], the total noncovalent interactions and grid sizes were 63 and 87 (Fig. 1A), respectively so that the systematic thermal instability was 1.38. With the same open state as a control, the calculated mean strucutral thermosensitivity $\left(\Omega_{10}\right)$ was about 21.0, which was close to the experimental $Q_{10}$ of 21.9 [13].

Clearly, the tight interaction of rTRPV1 with MSP2N2 allowed the E397-K710 H-bond in the pre-S1/TRP interface to decrease the conformational entropy and to inactivate the channel from a closed state below 53°C. Of special note, the smallest Grid_0_ also appeared in the same interface. It had a 0-residue size to control the H410-I696 π interaction and the D411-N695 H-bond via the shortest path from H410 to I696, N695, D411 and back to H410 (Fig. 1A, C & D). Since N695 and I696 are close to the lower gate [13], this Grid_0_ may play a critical role in inactivating rTRPV1 below 53°C.

### 3.2. Identification of an Inactivating Motif in Closed PI-free rTRPV1 at 48°C after 30 s

**Figure 2:**
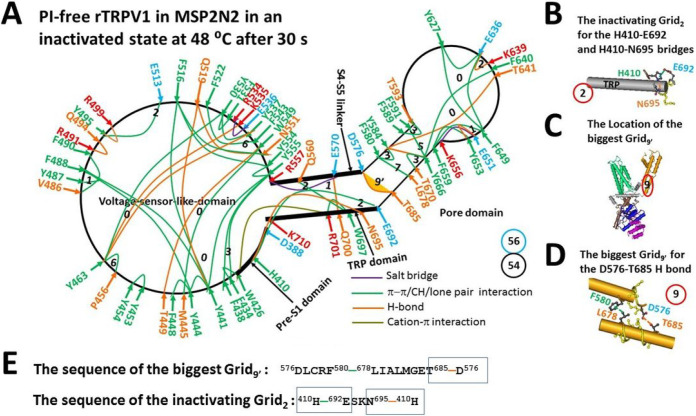
The grid-like non-covalently interacting mesh network along the PI-dependent gating pathway of PI-free rTRPV1 in an inactivated state at 48 °C. **A,** The topologocal grids in the systemic fluidic grid-like mesh network. The cryo-EM structure of one subunit of rTRPV1 with capsaicin bound at 48 °C for 30 s (EMD 23478; PDB ID, 7LPD) was used as a model for an inactivated state. The pore domain, the S4-S5 linker, the TRP domain, the VSLD and the pre-S1 domain are indicated in black. Salt bridges, p interactions, and H-bonds between pairing amino acid side chains along the PI-dependent gating pathway from D388 to K710 are marked in purple, green, and orange, respectively. The grid sizes required to control the relevant non-covalent interactions were calculated with graph theory and labeled in black. The D576-T685 H-bond in the biggest Grid_9_, was highlighted in yellow. The total grid sizes and grid size-controlled non-covalent interactions along the gating pathway are shown in the cyan and black circles, respectively. **B,** The inactivating Grid_2_ to govern the E692-H410-N695 bridges. **C,** The location of the biggest Grid_9_. **D,** The structure of the biggest Grid_9_ at the S5/S6 interface to control the D576-T680 H-bond. **E,** The sequences of the biggest Grid_9_, and the inactivating Grid_2_ with 9- and 2-residue sizes to control the D576-T680 and E692-H410-N695 bridges in the blue rectangles, respectively.

Along with PI-free rTRPV1 open at 48°C, the minor class for 30 s (EMD 23478, PDB-7LPD) was reported as a pre-open closed state because it was similar to the major class for 10 s (EMD 23477) [13]. However, when the total non-covalent interactions and grid sizes along the PI-dependent gating pathway were 54 and 61, respectively (Fig. 2A, Table S2), the calculated systematic thermal instability was actually 1.04, which was much smaller than that of the open state (1.65) (Table 1) [18]. Thereby, this very stable minor class could not be a pre-open state.

In addition, the smaller Grid_2_, which was similar to the inhibitive Grid_0_ in the pre-S1/TRP interface of PI-bound rTRPV1, was also present in the same interface to lock the channel closed (Figs. 1A & 2A). It had a 2-residue size to control the H410-E692 π interaction and the H410-N695 H-bond via the shortest path from H410 to E692, N695 and back to H410 (Figs. 2A, B & E). When two equivalent H-bonds sealed it, the calculated $T_{m}$ was about 70°C. Therefore, it could not be spontaneously melt at 48°C to generate an open state.

Finally, the biggest Grid_9′_, in the pore domain of the open state also appeared in this minor class after 30 s [18]. It had a 9-residue size to control the D576-T685 H-bond and the F580-L678 π interaction at the S5/S6 interface via the shortest path from D576 to F580, L678, T685 and back to D576 (Figs. 2A & C–E). In this case, the calculated $T_{m}$ of 61°C prevented it from being melt at 48°C to release the inhibitive Grid_2_ for channel opening (Table 1). More importantly, this biggest Grid_9′_, is a necessary anchor to keep the intact active pore domain of all the gating states including the open state [18]. In that regard, it is not allowed to be melt.

**Figure 3:**
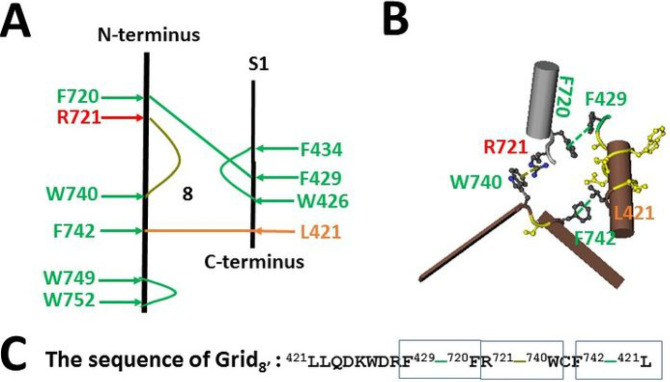
The grid-like non-covalently interacting mesh network between N- and C-terminal domains of Pl-free rTRPV1 beyond the PI-dependent gating pathway in an inactivated state at 48 °C. **A,** The topologocal grids in the systemic fluidic grid-like mesh network beyond the PI-dependent gating pathway at 48 °C. The cryo-EM structure of one subunit of rTRPV1 with capsaicin bound at 48 °C for 30 s (EMD 23478; PDB ID, 7LPD) was used as a model for an inactivated state. The N- and C-terminal domains are indicated in black along with S1. Several p interactions between pairing amino acid side chains from L421 to W752 are marked in green. The grid size required to control the relevant non-covalent interactions was calculated with graph theory and labeled in black. **B,** The structure of the biggest Grid_8′_ in the interface between N- and C-terminal domains to control the L421-F742 and F429-F720 and R721-W740 p interactions. C, The sequence of the biggest Grid_8′_ to control three p interactions in the blue rectangles.

When the search for the interactions between the N- and C-terminal domains was extended beyond the PI-dependent gating pathway, the F429-F720 and L421-F742 π interactions were found together with the R721-W740 cation-π interaction in the C-terminus (Fig. 3). In that case, although the second biggest Grid_8′_ was produced with an 8-residue size to control those three π interactions via the shortest path from L421 to F429, F720, R721, W740, F742, and back to L421, the predicted $T_{m}$ was still as high as 66°C. Accordingly, it could not be melt at 48°C to release the inhibitive Grid_2_.

Taken together, neither the inhibitive Grid_2_ could be dissociated below 61°C nor the biggest Grid_9′_, or Grid_8_ could be melt below 61°C to release the inhibitive Grid_2_. Thereafter, the minor class after 30 s was factually an inactivated state rather than a pre-open state. Moreover, when the open state was employed as a control, the calculated apparent mean structural thermo-sensitivity $\left(\Omega_{10}\right)$ was about − 7.60, which was actually higher than the measured $Q_{10}$ of −5.09 (Table 1). Thus, this inactivated state may originate not from the open state but from the pre-open closed state.

### 3.3. The Real Pre-open Closed State Was Different than the Inactivated State Structurally

**Figure 4:**
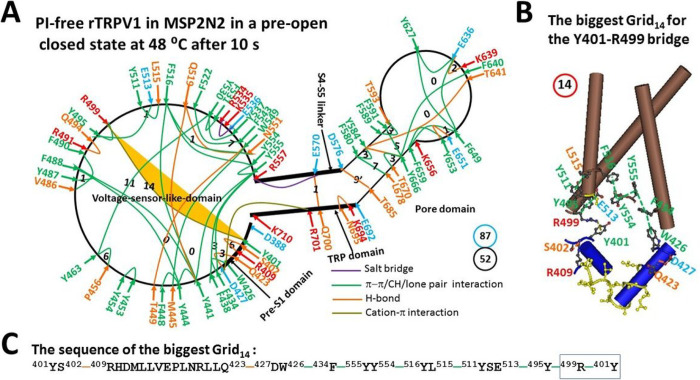
The grid-like non-covalently interacting mesh network along the PI-dependent gating pathway of PI-free rTRPV1 in a pre-open closed state at 48 °C. **A**, The topologocal grids in the systemic fluidic grid-like mesh network. The cryo-EM structure of one subunit of rTRPV1 with capsaicin bound at 48 °C for 10 s (EMD 23477) was used as a model for a preopen closed state. The pore domain, the S4-S5 linker, the TRP domain, the VSLD and the pre-S1 domain are indicated in black. Salt bridges, p interactions, and H-bonds between pairing amino acid side chains along the PI-dependent gating pathway from D388 to K710 are marked in purple, green, and orange, respectively. The grid sizes required to control the relevant non-covalent interactions were calculated with graph theory and labeled in black. The Y401-R499 p interaction in the biggest Grid_14_ was highlighted in yellow. The total grid sizes and grid size-controlled non-covalent interactions along the gating pathway are shown in the cyan and black circles, respectively. **B**, The structure of the biggest Grid_14_ in the pre-S1/VSLD interface to control the Y401-R499 p interaction. C, The sequence of the biggest Grid_14_ with a 14-residue size to control the Y401-R499 p interaction in the blue rectangle.

Further studies showed that the pre-open closed state was actually different than the inactivated state structurally (Fig. 4A). First, when the E692-H410-N695 bridges in the pre-S1/TRP interface were disrupted, the S402-R409 and Q423-D427 H-bonds were formed. Second, in the S4-S5 linker/TRP interface, when Q560-W697-R701 π interactions were broken, K694 H-bonded with N695. Third, in the outer pore loop, the E651-K656 salt bridge was disconnected. Finally, when Y401 in the pre-S1 domain formed the cation-π interaction with R499 in the VSLD upon the broken K535-Y463-Y537 H-bonds, the Y511-L515 π interaction appeared and the Y495-R499 H-bond became a π interaction. In this case, although both states share a similar structure, those differences resulted in the biggest Grid_14_ in the pre-S1/VSLD interface (Fig. 4A). It had a 14-residue size to control the Y401-R499 bridge via the shortest path from Y401 to S402, R409, Q423, D427, W426, F434, Y555, Y554, Y516, L515, Y511, E513, Y495, R499 and back to Y401 (Fig. 4B–C). When 1.5 equivalent H-bond sealed it, the calculated $T_{m}$ was about 41°C (Table 1). As the total noncovalent interactions and grid sizes were 52 and 87, respectively, the calculated mean $\Omega_{10}$ was 12.4, which was smaller than the experimental $Q_{10}$ of 16.4 for oocytes-expressing rTRPV1 without PI (Table 1). Meanwhile, the systematic thermal instability $\left(T_{i}\right)$ was 1.67, which was similar to that in the open state (Table 1). Accordingly, once the biggest Grid_14_ was dissociated, this pre-open state would quickly transit to the open state at the similar energy level.

### 3.4 The Inhibitive Grid_0_ in PI-Bound rTRPV1 Was Released upon the Removal of PI by Capsaicin at 4°C

**Figure 5:**
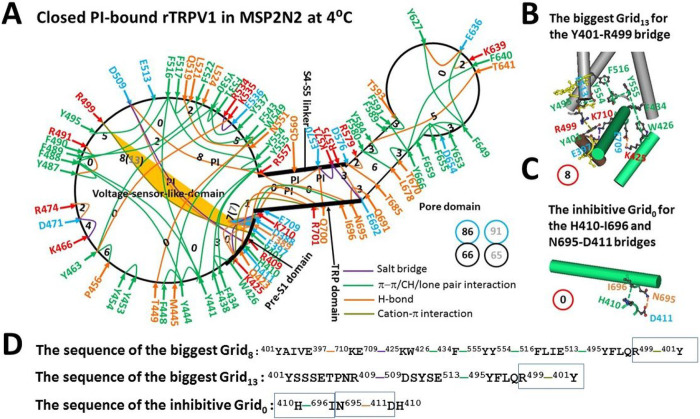
The grid-like non-covalently interacting mesh network along the PI-dependent gating pathway of PI-bound rTRPV1 in the closed state at 4 °C. **A,** The topological grids in the systemic fluidic grid-like mesh network. The cryo-EM structure of one subunit in closed rTRPV1 with PI bound at 4 °C (PDB ID, 7LP9) was used for the model. The pore domain, the S4-S5 linker, the TRP domain, the VSLD and the pre-S1 domain are indicated in black. Salt bridges, p interactions, and H-bonds between pairing amino acid side chains along the PI-dependent gating pathway from D388 to K710 are marked in purple, green, and orange, respectively. The grid sizes required to control the relevant non-covalent interactions were calculated with graph theory and labeled in black. The Y401-R499 bridge in the biggest Grid_13_ was highlighted in yellow. The total grid sizes and grid size-controlled non-covalent interactions along the PI-dependent gating pathway are shown in the cyan and black circles, respectively. When the inhibitive E397-K710 H-bond, which was highlighted in blue, was removed in the native lipid system, the putative grid sizes are labeled in grey. **B,** The structure of the biggest Grid_8_ with an 8-atom size for the Y401-R499 cation-p interaction near PI. **C,** The structure of the inhibitive Grid_0_ with a 0-residue size in the TRP/pre-S1 interface to control the H410-I696 and D411-N695 bridges near PI. **D,** The sequences of the biggest Grid_8_, Grid_13_ and Grid_0_ with 8-, 13- and 0-residue sizes to control the relevant bridges in the blue rectangles, respectively.

In the absence of the Y401-D707 H-bond but the presence of the E397-K710 H bond, PI-bound rTRPV1 at 4° C also had the same biggest Grid_8_ to control the Y401-R499 π interaction in the pre-S1/VSLD interface, and the same inhibitive Grid_0_ in the pre-S1/TRP interface to control the H410-I696 and D411-N695 bridges (Fig. 5). In that case, unless the broken E397-K710 H bond allowed the same biggest Grid_13_ to have the normal $T_{m}$ of 43°C for channel opening, the biggest Grid_8_ still increased the calculated $T_{m}$ up to 53°C to prevent both the PI lipid and the inhibitive Grid_0_ from releasing in the pre-S1/TRP interface for channel activation at 43°C. Since the total noncovalent interactions and grid sizes were 66 and 86, respectively (Table 1), if the channel opened above 53°C, the calculated mean $\Omega_{10}$ would be 21.8 (Table 1).

**Figure 6:**
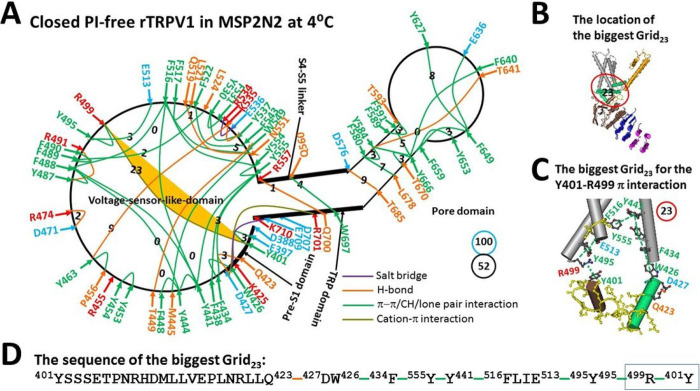
The grid-like non-covalently interacting mesh network along the PI-dependent gating pathway of capsaicin-bound rTRPV1 in the closed state at 4 °C. **A,** The topological grids in the systemic fluidic grid-like mesh network. The cryo-EM structure of one subunit in closed rTRPV1 with capsaicin bound at 4 °C (PDB ID, 7LPA) was used for the model. The pore domain, the S4-S5 linker, the TRP domain, the VSLD and the pre-S1 domain are indicated in black. Salt bridges, p interactions, and H-bonds between pairing amino acid side chains along the PI-dependent gating pathway from D388 to K710 are marked in purple, green, and orange, respectively. The grid sizes required to control the relevant non-covalent interactions were calculated with graph theory and labeled in black. The Y401-R499 bridge in the biggest Grid_23_ was highlighted. The total grid sizes and grid size-controlled non-covalent interactions along the PI-dependent gating pathway are shown in the cyan and black circles, respectively. **B,** The location of the biggest Grid_23_ in the pre-S1/VSLD interface. **C,** The structure of the biggest Grid_23_ with a 23-residue size in the pre-S1/VSLD interface to control the the R499-Y401 p interaction. **D,** The sequence of the biggest Grid_23_ to control the R499-Y401 p interaction in the blue box.

When capsaicin competes off the PI lipid from the vanilloid site in PI-free rTRPV1 at 4°C, the biggest Grid_23_ was present to control the Y401-R499 π interaction in the pre-S1/VSLD interface (Fig. 6). When two equivalent H-bonds sealed it, the calculated $T_{m}$ was about 28°C (Table 1). If the channel opened from this closed state above 28°C, the total noncovalent interactions of 52 and grid sizes of 100 would generated the systematic thermal instability $\left(T_{i}\right)$ as 1.92 and the mean $\Omega_{10}$ as 25.4 (Table 1). Of special note, the inhibitive Grid_0_ in the pre-S1/TRP interface was released. However, the inhibitive Grid_2_ in the inactivated state of Pl-free rTRPV1 at 48°C was absent in the closed state of Pl-free rTRPV1 at 4°C (Figs. 2A & 6A, Table 2). In that regard, the inhibitive Grid_2_ at 48°C was factually formed by the heat stimulus rather than capsaicin. This finding was consistent with the notion that capsaicin and heat desensitizes TRPV1 through a distinct structural basis [12].

## 4. Discussion

The TRPV1 bio-thermometer takes a critical role in detecting noxious heat and protecting human or animal bodies from heat damage [1, 24–26]. Recently, the graphical grid thermodynamic model has successfully been used to identify not only five biggest grids in TRPV1 as the specific local thermoring sensors for different gating states but also some smaller grids for heat efficacy. In this computational study, this graphical method was further exploited to uncover the structural factors or motifs for not only the inactivation from the open state regarding the optimal and maximal activity temperatures but also the inactivation from the pre-open closed state regarding the use-dependent desensitization. Taken together, this complex inactivation may play a critical role in alleviating pain upon constant or repeated noxious heat stimuli.

### 4.1 Thermal Inactivation from the Open State for the Maximal Activity Temperature of 61°C.

Once PI was released from the active vanilloid site of rTRPV1, Grid_9′_, was highly conserved in all the gating states including the open one. In fact, the residues in this grid are also highly conserved in TRPV1-6 [27]. It had a 9-residue size to control the D576-T685 H-bond and the F580-L678 π interaction at the S5/S6 interface via the shortest path from D576 to F580, T685, and back to D576 (Figs. 2A, C & D). When 2.5 equivalent H-bonds sealed this Grid_9′_, the calculated $T_{m}$ was about 61°C, which was exactly the same as the maximal activity temperature of rTRPV1 [3]. To this end, Grid_9′_, was a necessary anchor or heat fuse to keep the channel functional. Any thermostable perturbation around it may trigger the partial thermal inactivation of the ion conduction pathway from an open state, causing flickering opening [15, 28]. Furthermore, any mutation like R579E/A or D576N/R in rTRPV1 or T680A in mTRPV3 (T685 in rTRPV1) in this Grid_9′_, leaves the channel non-functional or insensitive to heat [29, 30]. Therefore, Grid_9_ may be the thermoring for the maximal activity temperature 61°C [3].

### 4.2 Thermal Inactivation from the Open state for the Optimal Activity Temperature of 56°C.

In addition to Grid_9′_, there is another biggest Grid_9_ in the open state of rTRPV1 [18]. It had a 9-residue size to control the stimulatory R557-E570 bridge via the shortest path from R557 to E570, Q560, W697, R701, Q700, and back to R557 [18]. When 2 equivalent H-bonds sealed it, the calculated $T_{m}$ is 56°C, at which the channel activity is maximal [2, 3]. When the temperature raised above 56°C, the weakened R557-E570 bridge may also allow thermal inactivation from the open state [3]. In agreement with this proposal, the R557E or E570L mutant has no function, or mutants R557A/L and E570A or nearby M572A are insensitive to heat [13. 29, 31]. In that regard, Grid_9_ at the VSLD/S4-S5 linker/TRP interfaces of the open state may be responsible for the optimal activity temperature 56°C.

### 4.3 Thermal Inactivation from the Pre-open Closed State for the Use-dependent Desensitization between 43°C and 61°C.

**Figure 7:**
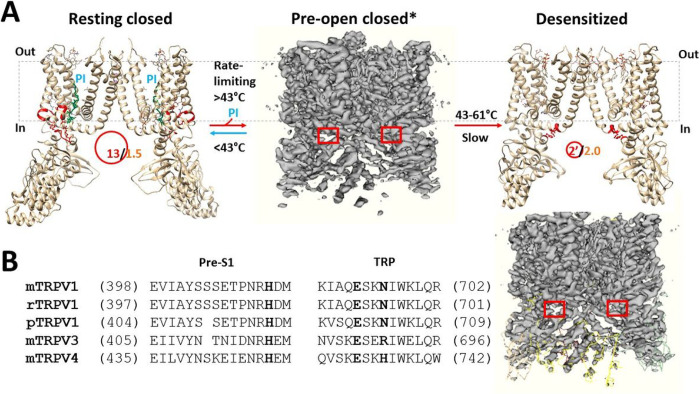
The tentative model for rTRPV1 inactivation from the pre-open closed state upon the heat-evoked melting of the biggest grid. The homo-tetrameric cryo-EM structures of rTRPV1 in the resting closed, inactivated, and pre-open closed states (PDB ID: 7LP9, 7LPD/EMD-23478 and EMD-23477, respectively) were used for the model. For a convenient view, only two opposite subunits are shown for the resting closed and inactivated states. The dashed rectangles are the membrane areas. In the presence of the PI lipid (blue) at the active capsaicin site, rTRPV1 has 1.5 equivalent H-bonds (orange) to seal the biggest Grid_13_ (red) with a 13-residue size (red) in the VSLD/pre-S1 interface so that rTRPV1 is closed below a threshold 43 °C. When the temperature increases above the threshold 43 °C to remove the PI lipid from the active vanillpoid site, the lower $T_{i}$ (1.04) and the smaller Grid_2_ (red) in the pre-S1/TRP interface may allow rTRPV1 to bypass the open state and to inactivate slowly from the pre-open closed state at prolonged temperature between 43 °C and 61 °C in an irreversable manner. **B,** The sequence alignment of TRPV1-4 along the pre-S1 domain and the TRP domain.

In addition to the thermal inactivation from the open state above 56°C until 61°C, the minor class at 48°C after 30 s shares the similar 3D structure with the major class at 48°C after 10 s [13]. This similarity reminded the inactivation of the Kv4 channel from the pre-open closed state [32]. Therefore, once the latter serves as the pre-open closed state, it would be followed by the inactivated state between 43°C and 61°C for several reasons (Figs. 2A & 7A). First, the experimental $T_{\text{th}}$ (> 41°C) for rTRPV1 activation and use-dependent irreversible desensitization was similar [2–4]; Second, the biggest Grid_14_ in the pre-open closed state has been replaced with the biggest Grid_9′_ in the inactivated state (Figs. 2A and 4A). Thus, it could survive between 43°C and 61°C; Third, the stimulatory R557-E570 H-bond controlled by the biggest Grid_9_ in the open state was governed by the smaller Grid_1_ in the inactivated state via the shortest path from R557 to E570, Q700, R701, W426, F434, Y555 and back to R557. When five equivalent H-bonds sealed it, the calculated $T_{m}$ was up to 102°C. On the other hand, the inhibitive Grid_2_ in the pre-S1/TRP interface had a $T_{m}$ of 70°C. It was not present in the open state at 48°C and the closed state at 4°C once the PI lipid was released from the vanilloid site (Fig. 6, Table 2) [18]. Hence, this inactivated state was irreversible between 43°C and 61°C; Fourth, the calculated apparent mean $\Omega_{10}$ from the open state to this inactivated one was −7.60, which was higher than the experimental $Q_{10}$ (−5.09) (Table 1). Thereby, the inactivated state may originate from the same pre-open closed state rather than the open state.

It should be noteworthy that the similar systematic thermal instability (Ti, ~1.66) between the closed state and the open state of Pl-free rTRPV1 promotes the fast channel opening within 30 s (Table 1) [18]. In contrast, the dramatic decrease of this parameter from 1.67 to 1.04 slowed down the inactivation from the same pre-open closed state (Table 1). That may be why the minor class appeared after 30 s [6], and would become a major one after 50 s [4].

It is interesting that the sequence alignment demonstrated that the missing serine between E404 (E397 in rTRPV1) and H416 (H410 in rTRPV1) of pTRPV1 or the substitution of N695 in rTRPV3 by R690 in mTRPV3 may prohibit the formation of the inhibitive Grid_2_ in the pre-S1/TRP interface (Fig. 7B) [27, 33]. This difference may account for the absence of the use-dependent desensitization when the N- and C-terminal domains of rTRPV1 are replaced by those aligned parts of pTRPV1 or the C-terminal of rTRPV1 is substituted by the aligned part of mTRPV3 [4–12]. On the other hand, rTRPV1 or mTRPV1 shares the similar pre-S1 domain and TRP domain with mTRPV4 (Fig. 7B). Thus, TRPV4 may have the same inactivation pathway as rTRPV1 used for the use-dependent heat desensitization [34–35]. In addition, Unlike HEK293 cell-expressing rTRPV1, oocytes expressing rTRPV1 exhibits minimal desensitization of heat-evoked responses [36], possibly by weakening the inhibitive E692-H410-N695 bridges in the different membrane system. Further structural and functional measurements are necessary to test the role of the smaller Grid_2_ or the E692-H410-N695 bridges in the use-dependent desensitization between 43°C and 61°C

## 5. conclusion

The graph theory-based grid thermodynamic model has been successfully used to define the structural motifs for the activation and the inactivation of biological globular proteins such as class I or II fructose aldolases. While the melting of the biggest grid near the active site is generally required for the activation, the thermal unfolding of the anchor grid is necessary for the inactivation. In this computational study, two biggest grids in the open state of rTRPV1 were required to be melt for the inactivation above the optimal activity temperature until the maximal activity temperature. Although the smaller grid in the pre-S1/TRP interface inhibited the rTRPV1 activity in both closed and inactivated states, a lower threshold was enough to release the inhibitive grid in the closed state for channel opening. In contrast, a higher threshold was needed to remove it, resulting in the irreversible inactivation from the pre-open closed state below that threshold. These two different thermal inactivation pathways may provide new insights into the parallel world in the active bio-macromolecules.

## Figures and Tables

**Table 1:** New parameters of the rTRPV1 bio-thermometer based on the grid thermodynamic model

Construct	Control		rTRPV1				
**Lipid environment**	MSP2N2		MSP2N2				
**PDB ID or EMD #**	7LPE	7LPE	7LPC	23477	7LPD	7LP9	7LPA
**Lipid PI at the capsaicin site**	free		bound	free	free	bound	free
**Sampling temperature, °C**	**48**		**48**	**48**	**48**	**4**	**4**
**Gating state**	open		closed	pre-open closed	inactivated	closed	closed
**Name of the biggest grid**	Grid_9_	Grid_9'_	Grid_8_	Grid_14_	Grid_9'_	Grid_8_	Grid_23_
**Biggest grid size (S_max_)**	9	9'	8	14	9'	8	23
**Equivalent H-bonds in S_max_**	2.0	2.5	1.5	1.5	1.5	1.5	2.0
**Total non-covalent interactions**	43		64	52	54	66	52
**Total grid sizes, a.a./atoms**	71		82	87	56	86	100
**Systematic thermal instability (T_i_)**	1.65		1.28	1.67	1.04	1.30	1.92
**Calculated T_m_°C**	**56**	**61**	**53**	**41**	**61**	**53**	**28**
**Measured threshold T_th_, °C**	**56**	**61**	**>48**	**42**	**61**	**>48**	**<48**
Calculated Ω_10, min_ at E = 0.5 kJ/mol			4.51	5.35	−3.17	7.56	11.0
**Calculated Ω_10, mean_ at E = 1.0 kJ/mol**			**12.7**	**12.4**	**−7.60**	**21.8**	**25.4**
Calculated Ω_10, max_ at E = 3.0 kJ/mol			65.2	46.8	−30.3	117	96.1
**Measured Q_10_**			**21.9**	**16.4**	**−5.09**	**21.9**	**16.4**
**References for T_th_and Q_10_**	[2, 3]	[3]	[13]	[2]	[3]	[13]	[13]

**Table 2:** Noncovalent interactions between N and C termini of rTRPV1 in the presence or absence of the stimulatory R557-E570 bridge between S4b and the S4-S5 linker.

PDB ID	7LPD	7LPE	7LPC	7LPB	7LPA	7LP9
ligand	Cap	Cap	PI	Cap	Cap	PI
Sampling T	48 °C	48 °C	48 °C	25 °C	4 °C	4 °C
gating	inactivated	open	closed	closed	closed	closed
R557-E570	+	+	−	−	−	−
E397-K710	−	−	+	−	−	+
H410-E692	+	−	−	−	−	−
H410-I696	−	−	+	−	−	+
H410-N695	+	−	−	−	−	−
W426-R701	+	+	+	+	+	+
F429-F720	+	−	+	+	+	+
L421-F742	+	−	−	−	−	−

Note: C terminal region, 687N-838K; N-terminal region, 1M-432R.

## Data Availability

All data generated or analysed during this study are included in this published article. Tables S1-S5 are available in the Supplementary Information.

## Abbreviations

Cap: capsaicin
cryo-EM: cryo-electron microscopy
$\Omega_{10}$: structural thermosensitivity
$Q_{10}$: functional thermo-sensitivity
PI: phosphatidylinositol
$P_{0}$: open probability
RTx: resiniferatoxin
$T_{i}$: systematic thermal instability
$T_{m}$: melting temperature threshold
$T_{\text{th}}$: temperature threshold
TRP: transient receptor potential
TRPV1: TRP vanilloid-1
TRPVi: (i=1; 2, 3, 4, 5, 6)
hTRPV1: human TRPV1
mTRPV1: mouse TRPV1
mTRPV3: mouse TRPV3
mTRPV4: mouse TRPV4
pTRPV1: platypus TRPV1
rTRPV1: rat TRPV1
VSLD: voltage-sensor-like domain
